# Health and well-being of older adults in rural and urban Rwanda: epidemiological findings from a population based cross-sectional study

**DOI:** 10.7189/jogh.15.04108

**Published:** 2025-05-05

**Authors:** Michael Boah, Callixte Cyuzuzo, Francois Uwinkindi, Chester Kalinda, Tsion Yohannes, Sandra Isano, Carolyn Greig, Justine Davies, Lisa R Hirschhorn, Alemayehu Amberbir

**Affiliations:** 1 University of Global Health Equity, Centre for Population Health, Kigali, Rwanda; 2 Ministry of Health, Rwanda Biomedical Center, Kigali, Rwanda; 3 University of Global Health Equity, Bill and Joyce Cummings Institute of Global Health, Kigali, Rwanda; 4 University of Global Health Equity, Centre for Gender Equity, Kigali, Rwanda; 5 University of Global Health Equity, Department of Community Health and Social Medicine, Kigali, Rwanda; 6 University of Birmingham, School of Sport, Exercise and Rehabilitation Sciences, Birmingham, UK; 7 University of Birmingham, Institute of Applied Health Research, Birmingham, UK; 8 Feinberg School of Medicine, Northwestern University, Chicago, Illinois, USA

## Abstract

**Background:**

Ageing often leads to multimorbidity, frailty, and disability; these interconnected conditions significantly impact quality of life (QoL) and strain healthcare systems through increased dependency and care needs. Despite their importance for health system planning, they remain understudied in Rwanda's older population. Here we describe the epidemiology of these outcomes in Rwanda’s ageing population.

**Methods:**

We conducted a cross-sectional, population-based study among Rwandan adults aged ≥40 years across urban and rural districts, whereby we used validated tools to assess multimorbidity (≥2 chronic conditions), frailty (Fried Frailty Score), disability (World Health Organization Disability Assessment Schedule (WHODAS) 2.0), and QoL (European Health Interview Survey – World Health Organization Quality of Life (EUROHIS-QoL)). We used multivariable analyses to examine associations between the outcomes and demographic and socioeconomic factors.

**Results:**

Among 4369 adults, multimorbidity prevalence was 55.2% (95% confidence interval (CI) = 53.7, 56.6), with frailty affecting 14.5% (95% CI = 13.5, 15.6) of this population. Disability prevalence was relatively low, with a median score of 10.4% (interquartile range = 2.1–25.0), while the mean QoL score was 48.2% (standard deviation = 15.6). We observed impairment in activities of daily living (ADL) in 16.0% (95% CI = 14.9, 17.1) of the sample. Health outcomes worsened with age, particularly among those aged ≥70 years, and among females compared to males. Multivariable analyses showed that higher socioeconomic status and urban residence were significantly associated with lower frailty, disability, and ADL impairment, though urban residents had higher multimorbidity rates and poorer QoL. Higher educational status was associated with reduced disability and improved QoL.

**Conclusions:**

Our findings show a substantial burden of multimorbidity and frailty among older adults in Rwanda, with significant gender, socioeconomic, and urban-rural disparities. Integrated care models that address both the physical and social determinants of health, with a focus on reducing gender, socioeconomic, and geographical disparities, are needed to improve the well-being of older adults in Rwanda.

The global population of older adults (typically defined as individuals aged ≥60 years) is rapidly increasing, particularly in sub-Saharan Africa (SSA), where the number is projected to increase significantly due to declining fertility and increasing longevity [1]. These demographic changes in SSA have been attributed in part to advances in public health, medical interventions, and socioeconomic development [2–4]. However, with longer life spans, individuals are also more likely to experience multiple chronic conditions simultaneously, leading to multimorbidity [5,6].

Multimorbidity, defined as the co-existence of two or more chronic conditions, has emerged as a significant public health concern, with a pooled prevalence of 42%, according to a systematic review and meta-analysis of data from 193 international studies [7]. The prevalence also varies widely, particularly in low- and middle-income countries (LMICs).For example, some studies in these regions have found that over 80% of people aged ≥60 years are affected [5,6]. Various factors such as urbanisation, lifestyle changes, and demographic shifts account for the increasing prevalence of chronic conditions in LMICs [8,9]. Unfortunately, multimorbidity is complicated to manage and increases the risk of adverse health outcomes, including functional decline and decreased quality of life (QoL) [10–12]. The situation is particularly dire in SSA, where fragmented healthcare systems, resource constraints, and high rates of polypharmacy pose significant challenges to patient care [13,14]. Additionally, social determinants, such as poverty and limited education, further compound health disparities, while stigma surrounding mental health prevents the effective management of comorbidities [15].

Frailty is a common condition in ageing populations [16,17]. Individuals with multimorbidity often experience frailty, characterised by declines in physical function, as well as increased vulnerability to stressors and adverse health outcomes [18–20]. The combination of multimorbidity and frailty significantly impacts an individual's ability to perform daily activities, leading to increased rates of disability [21]. This decline in functional ability not only affects physical health but also diminishes QoL, as individuals may face limitations in social participation and independence [22]. The economic impact of frailty and disability is particularly pronounced in LMICs, where healthcare systems are often under-resourced and ill-equipped to manage chronic conditions [23]. The increased demand for medical care, hospitalisations, and long-term support places significant financial strain on both individuals and national health budgets [24,25]. Additionally, the inability to engage in economic activities due to poor health exacerbates poverty, creating a cycle of financial and healthcare challenges.

Over the last three decades, Rwanda has made considerable progress in healthcare, with significant declines in under-five mortality and HIV control [26,27]. These achievements stem from a strong commitment to universal health coverage, community-based health insurance (*Mutuelles de Santé*), and a focus on primary healthcare and preventive services. However, the country now faces an emerging challenge of addressing age-related conditions, as life expectancy has seen a dramatic increase from 46 years in 1978 to approximately 70 years in 2022 [28]. Like many other African countries, Rwanda’s healthcare system has primarily focussed on maternal and child health, as well as communicable diseases, while efforts to understand and address the burden of multimorbidity and frailty among older adults remain largely underexplored [29]. Despite efforts to integrate noncommunicable diseases services into primary healthcare, significant gaps persist in geriatric care. Many healthcare facilities lack the capacity to provide specialised services for older adults, and a substantial portion of healthcare workers remain untrained in geriatrics [30]. So, while the country continues to strengthen its healthcare system, understanding the burden of health outcomes, including multimorbidity, frailty, and disability, is essential for the development of age-friendly policies and for achieving universal health coverage. Moreover, Rwanda’s unique historical and demographic context, including the effects of trauma during and after the Rwandan genocide and rapid socioeconomic changes, may contribute to distinctive patterns of health outcomes in its ageing population (including frailty and disability) due to the social and psychological impacts of these events [31,32].

However, data on the burden of multimorbidity, frailty, and disability among older adults in this setting remain limited. We sought to fill this gap by describing the epidemiology of these conditions in Rwanda and quantifying their associations with individual characteristics, so as to provide evidence to design novel interventions for maintaining healthy ageing and inform policy and planning in the study setting.

## METHODS

### Study design and settings

Rwanda is a low-income country where approximately 7% of the population is >60 years old, the retirement age is 65 years, and the life expectancy (as of 2022) is approximately 70 years [28]. We conducted this cross-sectional household survey in four districts of Rwanda: the three districts of the City of Kigali (Gasabo, Kicukiro, and Nyarugenge) represented urban areas, while Burera District in Northern Rwanda represented rural settings, as defined in the recent population and housing census [28].

### Study population

#### Age definition and eligibility criteria

We defined older adults as individuals aged ≥40 years, which is consistent with our previous studies in Africa [16,33]. This age threshold was selected since many countries in SSA have a low life expectancy and a limited population of individuals >60 years – an age typically used to define older adults in other countries. We have also found that the magnitude of frailty among people >40 years is significantly higher than would be expected in a high-income population of the same age [33,34]. To be eligible, respondents had to have resided primarily within a selected village in the study setting for at least one year before the survey and have been willing to consent to participate. We decided to apply a one-year residency criterion to ensure that participants had sufficient exposure to the local healthcare system and village environment, which is critical for assessing long-term health outcomes such as multimorbidity and frailty.

#### Sample size determination

We computed the sample size to detect a frailty prevalence using the Fried Frailty Score of 7% [16]. With a margin of error of +/−0.5%, a 95% confidence level, and accounting for a non-response rate of 10%, we estimated that we required at least 4280 respondents. We derived this sampling frame from the 2022 Population and Housing Census in Rwanda, with support from the National Institute of Statistics of Rwanda [28].

#### Sampling strategy

We applied a two-stage sampling technique to ensure representation of the target population. In the first stage, we selected 127 villages from the sampling frame using a probability proportional to size strategy for both urban and rural areas across the four districts. This method ensured that the larger districts contributed more participants, while smaller districts were represented proportionally. The study protocol is described in detail elsewhere [35]. Based on information from the most recent census [28], we selected a minimum of 30 households in each village, interviewing one eligible respondent per household. If there were multiple eligible individuals, we employed a lottery system to randomly choose one for the interview. Specifically, we assigned each eligible individual a unique identifier and selected one randomly, ensuring equal selection chances and reducing bias.

### Data collection and procedures

Trained data collectors collected data electronically into a web-based Research Electronic Data Capture (REDCap) between March and June 2024 using an Android operating platform. The data collection tool, a structured questionnaire, was adapted from one used in our previous studies in other countries in SSA and administered in Kinyarwanda [16,19,33,36]. Sex was captured as male or female. Respondents’ age was collected in continuous form and later divided into age intervals for analysis. Marital status was classified into three categories: single (never married), married or cohabiting (indicating a partnership), and previously married (divorced, separated, or widowed). The highest level of education completed was categorised as no formal education, primary, secondary, and tertiary education. Wealth quintiles were generated based on 18 questions covering household assets and dwelling characteristics (such as the source of drinking water, type of toilet facility, cooking fuel, and ownership of items like a television, computer, etc.) using the first principal component [37]. Rural or urban residence was captured as described in the fifth Rwanda Population and Housing Census Main Indicators Report [28].

Self-reported chronic medical conditions included hypercholesterolemia, heart disease, stroke, asthma, chronic obstructive pulmonary disease (COPD), cancer, musculoskeletal disorders (including bone and joint disorders), chronic eye conditions (such as cataracts or glaucoma), chronic kidney disease, and liver conditions. Weight loss was assessed using the question, ‘How much weight have you lost in kg over the last year?’ Self-reported exhaustion was measured using two questions from the eight-item Center for Epidemiologic Studies Depression (CES-D) scale: ‘Everything I did in the last week was an effort’ and ‘I could not get going’ [38]. These two questions were chosen because they specifically capture fatigue-related symptoms and have been previously used in the original Fried frailty derivation score and epidemiological research in SSA as proxies for exhaustion [34,39].

We assessed anxiety symptoms using the Generalised Anxiety Disorder two-item (GAD-2) questionnaire [40], cognitive functioning using the Community Screening Instrument for Dementia (CSI-D) [41], and depressive symptoms using the Patient Health Questionnaire (PHQ-9) [42], We assessed disability using the 12-item World Health Organization Disability Assessment Schedule (WHODAS) 2.0 [43]. The questions evaluated the individual's performance in daily activities over the past 30 days, covering six domains: cognition, mobility, self-care, getting along (dealing with people you do not know and maintaining friendship), life activities, and participation in society. We assessed quality of life using the eight-item European Health Interview Survey – World Health Organization Quality of Life (EUROHIS-QoL) scale, which covers psychological, physical, social, and environmental domains [44]. Information was gathered on the six basic activities of daily living (ADL) – walking, transfers, toileting, bathing, eating, and dressing – to evaluate the functional abilities of the respondents [45]. Response options were none, mild, moderate, severe, extreme, or unable to do.

We measured respondents’ weight once, to the nearest 0.1 kg, using the Omron HN289 digital personal scale, while height was measured with a tape measure. We measured participants’ blood pressure using the Omron HEM-7322 automatic digital device (OMRON Healthcare Co., Ltd, 245 Kyoto, JAPAN), whereby three readings were taken at five-minute intervals on the left arm, starting after a 15-minute rest. The average of the second and third readings was used for analysis [46]. We measured handgrip strength using the CAMRY hand dynamometer (CAMRY EH101, Sensun251 Weighing Apparatus Group Ltd, Guangdong, China). We assessed both hands two times, and the maximum value of the two hands was used in this analysis [16,34]. We measured walk speed by timing participants while walking a four-metre course at their usual pace. Specifically, we conducted two timed trials in opposite directions using a digital stopwatch, calculating walking speed (meters/s) for each direction and using the fastest speed adjusted for height for analysis. Lastly, we measured glucose concentrations from a finger-prick blood sample using an Accu-Chek (Roche Diabetes Care, India) glucose monitor [47].

### Outcomes

Outcomes included multimorbidity, frailty, disability, QoL, and impairment in ADLs. Multimorbidity was identified when individuals had two or more chronic conditions. In addition to self-reporting, we defined hypertension as a systolic pressure of ≥140 mm Hg or diastolic pressure of ≥90 mm Hg [48] and diabetes mellitus as a fasting glucose concentration >126 mg/dL or a non-fasting glucose concentration >200 mg/dL [49]. We defined anxiety symptoms as a GAD-2 score of ≥3 [40], a CSI-D score <6 as indicative of the presence of dementia [41], and depressive symptoms as a PHQ-9 score of 10 or more [42]. We grouped reported conditions into two domains: cardiometabolic (hypertension, heart diseases, stroke, diabetes, and hyperlipidaemia) and mental health conditions (anxiety, depressive symptoms, and cognitive impairment). We classified patients with multiple conditions within one domain as having concordant multimorbidity, and those with conditions across both domains as having discordant multimorbidity.

We used the Fried Frailty phenotype to assess frailty status [39] due to its ease of application and its frequent use in LMICs [50]. We determined scores using five domains: weight loss, low grip strength, low walking speed, self-reported exhaustion, and low activity levels [39]. We chose the thresholds for each domain to align as closely as possible with those used in the original Fried derivation criteria that have been employed in our previous study [34] and in other research [51]. Specifically, we defined weight loss as a self-reported loss of more than 4 kg over the past year [34,51]; low grip strength as the lowest quintile of body mass index (BMI)-adjusted grip for each sex (with BMI defined as respondents’ weight [kg] divided by the square of their height (m^2^)) [39]; low walk speed as the lowest quintile of height-adjusted walk speed [34]; self-reported exhaustion as answering that either of the two questions used applied for 3–4 days or 5–7 days per week [39]; and low physical activity as being in the highest quintile of self-reported hours of sitting per week reclining for each sex. Total frailty scores ranged from 0 to 5 points, with 0 categorised as non-frail, 1–2 as pre-frail, and 3 or more points as frail [39]. Some participants had missing data in one or more of the physical activity domains and were categorised as ‘unable to score’. However, prior research in an African context indicates that these individuals tend to have a health prognosis comparable to or worse than those identified as frail [19]. Consequently, we included these participants in the frail group for analysis.

We summed scores from the EUROHIS-WHOQoL scale (ranging from 8 to 40) and normalised them to a 0–100 scale, with higher scores indicating a better quality of life. We similarly summed and normalised scores from the WHODAS scale (ranging from 12 to 60) to a 0–100 scale, with higher scores reflecting greater disability. We defined impairment in any ADL as reporting any difficulty or inability to perform the activity. Among individuals with ADL impairments, we classified those reporting extreme difficulty or inability in any activity as having extreme impairment, those with moderate to severe difficulty as having moderate to severe impairment, and those reporting mild difficulty as having mild impairment.

### Statistical analysis

We performed all analyses using Stata/SE, version 18.0 (StataCorp LLC, College Station, Texas, USA). We summarised categorical variables as counts and percentages, and continuous variables as means and standard deviation (SD) for normally distributed data or medians with interquartile ranges (IQRs) for non-normally distributed ones. We assessed data normality using histograms and quantile-quantile (Q-Q) plots. We used chi-squared tests to compare categorical variables across groups. For non-normally distributed data, we used either the Mann-Whitney U test for two-group comparisons or the Kruskal-Wallis H test for three or more groups, with *post-hoc* pairwise comparisons using the Dunn test (with Bonferroni adjustment). We analysed normally distributed data using Student’s *t*-test (two groups) and analysis of variance (multiple groups), with Tukey's honest significant difference test identifying specific group differences. We analysed the associations between categorical outcomes (multimorbidity, frailty, and ADL impairment) and background characteristics using multivariable logistic regression, and continuous outcomes (disability and quality of life) using linear regression. We included characteristics that were statistically significant in bivariate analysis in the multivariable models, along with place of residence, which we added to account for its influence on health disparities and access to care. We assessed collinearity using the variance inflation factor test, which indicated no collinearity. We defined statistical significance as a *P* value <0.05.

### Ethics

The Rwanda National Ethics Committee (Reference no: RNEC262/2023), Northwestern University, USA (IRB ID: STU00220814), and the University of Birmingham, UK (IRB ID: ERN-23–0421) gave ethical approval for this study.

## RESULTS

Most participants (n = 3536, 80.9%) resided in urban areas, reflecting the sampling framework. Women accounted for nearly two-thirds of the sample (n = 2757, 63.1%). Age distribution showed that 1665 (38.1%) were aged 40–49, while 1528 (35%) were ≥60 years. In terms of education, 2001 (45.8%) individuals had completed primary education and 2822 (64.6%) were either married or cohabiting. These trends were largely consistent across rural and urban settings, except for education. In rural areas, 519 (62.3%) of respondents had no formal education, compared to urban areas, where 1248 (35.3%) had completed primary education (**Table 1**).

**Table 1:** Distribution of respondents by key demographic characteristics*

	Total (n = 4369)	Rural (n = 833, 19.1%)	Urban (n = 3536, 80.9%)	*P-*value†
**Age group in years, x̄ (SD)**	55.6 (12.2)	59.7 (12.9)	54.7 (11.8)	<0.001
40–49	1665 (38.1)	215 (25.8)	1450 (41.0)	
50–59	1176 (26.9)	203 (24.4)	973 (27.5)	
60–69	865 (19.8)	209 (25.1)	656 (18.6)	
≥70	663 (15.2)	206 (24,7)	457 (12.9)	
**Sex**				0.710
Female	2757 (63.1)	521 (62.5)	2236 (63.2)	
Male	1612 (36.9)	312 (37.5)	1300 (36.8)	
**Marital status**				<0.001
Married/cohabiting	2822 (64.6)	586 (70.3)	2236 (63.2)	
Single/divorced/separated/widowed	1547 (35.4)	247 (29.7)	1300 (36.8)	
**Educational level**				<0.001
No education	1767 (40.4)	519 (62.3)	1248 (35.3)	
Primary	2001 (45.8)	273 (32.8)	1728 (48.9)	
Secondary	491 (11.2)	41 (4.9)	450 (12.7)	
Tertiary	110 (2.5)	0 (0.0)	110 (3.1)	
**Wealth quintiles**				<0.001
Poorest	874 (20.0)	347 (41.7)	527 (14.9)	
Poorer	874 (20.0)	225 (27.0)	649 (18.4)	
Middle	886 (20.3)	191 (22.9)	695 (19.7)	
Richer	867 (19.8)	62 (7.4)	805 (22.8)	
Richest	868 (19.9)	8 (1.0)	860 (24.3)	

SD – standard deviation, x̄ – mean

*Presented as n (%) unless specified otherwise.

†χ^2^ test.

### Health and well-being status of respondents

Hypertension was the most prevalent condition, affecting 1751 (40.1%) individuals. Stroke affected 657 (15.0%), and musculoskeletal conditions affected 1277 (29.2%) individuals. Symptoms of anxiety were reported by 1395 (31.9%) and depression by 1018 (23.3%) individuals. Multimorbidity affected 2410 (55.2%; 95% confidence interval (CI) = 53.7, 56.6) individuals (**Table 2**; Table S1 in the **Online Supplementary Document**).

**Table 2:** Distribution of noncommunicable and chronic communicable disease diseases, multimorbidity, frailty, disability, quality of life, and impairment in activities of daily living

Categories, by outcome	Total (n = 4369)
Noncommunicable conditions	
*Hypertension*	1751 (40.1)
*High cholesterol*	43 (1.0)
*Raised blood glucose*	365 (8.4)
*Heart disease*	84 (1.9)
*Stroke*	657 (15.0)
*Chronic respiratory disease*	243 (5.6)
*Cancer*	69 (1.6)
*Musculoskeletal conditions*	1277 (29.2)
*Symptoms of anxiety on testing*	1395 (31.9)
*Depressive symptoms*	1018 (23.3)
*Symptoms of cognitive impairment*	500 (11.4)
Other NCDs*	827 (18.9)
Chronic communicable diseases	
*HIV infection*	308 (9.3)
Multimorbidity (any 2 or more chronic conditions)	
*No*	1959 (44.8)
*Yes*	2410 (55.2)
Frailty	
*Not frail*	1340 (30.7)
*Pre-frail*	2394 (54.8)
*Frail/unable to score*	635 (14.5)
Quality of life, x̄ (SD)	
*WHOQoL score (0–100%)*	48.2 (15.6)
Disability, MD (IQR)	
*WHODAS 2.0 score (0–100%)*	10.4 (2.1–25.0)
Impairment in ADL	
*None*	3671 (84.0)
*Some impairment (≥1 ADL)*	698 (16.0)
Impairment severity among individuals reporting impairment in ADL (n = 698)	
*Mild*	376 (53.9)
*Moderate to severe*	265 (38.0)
*Extreme*	57 (8.2)

ADL – activities of daily living, IQR – interquartile range, MD – median, NCD – noncommunicable disease, SD – standard deviation, WHODAS – World Health Organization Disability Assessment Schedule, WHOQoL – World Health Organization Quality of Life, x̄ – mean

*Other NCDs: chronic eye conditions (n = 757), chronic kidney diseases (n = 67), and chronic liver conditions (n = 28).

Over half of the population (n = 2394, 54.8%; 95% CI = 53.3, 56.3) were classified as prefrail, while 635 (14.5%; 95% CI = 13.5, 15.6) were considered frail, including 207 individuals with missing data due to incomplete physical measurements. The median disability score was 10.4% (IQR = 2.1–25.0), while the mean quality of life score was 48.2% (SD = 15.6). Among 698 (16.0%; 95% CI = 14.9, 17.1) individuals with ADL impairments, most (n = 376, 53.9%) reported mild impairment, with 265 (38.0%) experiencing moderate to severe impairments. The results showed that 124 (3.0%) individuals had probable dementia, while 376 (8.6%) showed signs of possible dementia. Regarding depression, 1444 (33.1%) individuals reported mild depressive symptoms. A smaller proportion experienced more severe forms: 254 (6.0%) showed moderately severe symptoms and 114 (3.0%) presented with severe depressive symptoms (Table S1 in the **Online Supplementary Document**). Chronic conditions and communicable diseases were generally more prevalent among females and urban residents, while rural residents showed higher rates of depression and cognitive impairment (Tables S1 and S2 in the **Online Supplementary Document**.

### Associations between multimorbidity, frailty status, disability, functional status (ADL), quality of life, and respondents’ background characteristics

#### Multimorbidity

The bivariable analysis showed that multimorbidity increased significantly with age and was more prevalent among females than males (Table S3 in the **Online Supplementary Document**). The multivariable analysis revealed that the odds of multimorbidity increased with age and were five times higher in the oldest age group (adjusted odds ratio (aOR) = 5.04; 95% CI = 4.04, 6.31). Males had lower odds of multimorbidity (aOR = 0.68; 95% CI = 0.60, 0.79). Being single, divorced, separated, or widowed was associated with increased odds of multimorbidity (aOR = 1.38; 95% CI = 1.19, 1.60). Higher wealth quintiles were linked to lower odds of multimorbidity, with the richest group experiencing the greatest benefit. Urban residents had significantly higher odds of multimorbidity compared to rural residents (aOR = 1.57; 95% CI = 1.32, 1.88). Education level did not impact multimorbidity (**Table 3**).

**Table 3:** Multivariable analyses of factors associated with multimorbidity, frailty status, disability, quality of life, and impairment in activities of daily of living

	Multimorbidity, aOR (95% CI)	*P*-value*	Frailty, aOR (95% CI)	*P*-value*	Disability score, β (SE)	*P*-value*	Quality of life score, β (SE)	*P*-value*	Impairment in ADLs, aOR (95% CI)	*P*-value*
**Age group in years**										
40–49	ref		ref		ref		ref		ref	
50–59	1.66 (1.42, 193)	<0.001	0.96 (0.74, 1.25)	0.772	4.85 (0.596)	<0.001	−1.61 (0.556)	0.004	2.66 (1.96, 3.60)	<0.001
60–69	2.94 (2.45, 3.52)	<0.001	2.13 (1.65, 2.75)	<0.001	11.93 (0.677)	<0.001	−3.13 (0.632)	<0.001	5.52 (4.09, 7.45)	<0.001
≥70	5.04 (4.03, 6.31)	<0.001	3.98 (3.06, 5.19)	<0.001	27.28 (0.773)	<0.001	−5.64 (0.722)	<0.001	19.57 (14.42, 26.55)	<0.001
**Sex**										
Female	ref		ref		ref		ref		ref	
Male	0.68 (0.59, 0.78)	<0.001	0.47 (0.37, 0.58)	<0.001	−4.03 (0.524)	<0.001	0.50 (0.489)	0.302	0.75 (0.60, 0.93)	0.008
**Marital status**										
Married or cohabiting	ref		ref		ref		ref		ref	
Single/divorced/separated/widowed	1.29 (1.11, 1.50)	0.001	1.05 (0.85, 1.29)	0.657	1.35 (0.563)	0.016	−1.06 (0.525)	0.044	1.08 (0.88, 1.34)	0.463
**Education level**										
No formal education	ref		ref		ref		ref		ref	
Primary	1.11 (0.96, 1.29)	0.166	0.86 (0.70, 1.07)	0.168	−0.67 (0.560)	0.232	0.46 (0.523)	0.384	0.92 (0.74, 1.15)	0.482
Secondary	1.18 (0.94, 1.49)	0.155	1.28 (0.91, 1.80)	0.157	1.32 (0.879)	0.133	−0.62 (0.821)	0.450	1.56 (1.12, 2.17)	0.009
Tertiary	0.76 (0.50, 1.17)	0.217	0.65 (0.27, 1.54)	0.324	−4.70 (1.623)	0.004	6.95 (1.516)	<0.001	0.41 (0.17, 1.00)	0.050
**Wealth quintiles**										
Poorest	ref		ref		ref		ref		ref	
Poorer	0.76 (0.62, 0.92)	0.010	0.78 (0.61, 1.01)	0.061	−2.96 (0.755)	<0.001	6.16 (0.705)	<0.001	0.82 (0.63, 1.07)	0.141
Middle	0.68 (0.55, 0.82)	<0.001	0.61 (0.46, 0.81)	<0.001	−5.21 (0.772)	<0.001	7.71 (0.721)	<0.001	0.58 (0.43, 0.77)	<0.001
Richer	0.65 (0.52, 0.82)	<0.001	0.76 (0.57, 1.02)	0.066	−5.63 (0.807)	<0.001	10.30 (0.754)	<0.001	0.54 (0.40, 0.75)	<0.001
Richest	0.63 (0.50, 0.72)	<0.001	0.51 (0.36, 0.71)	<0.001	−5.67 (0.865)	<0.001	15.22 (0.808)	<0.001	0.74 (0.54, 1.02)	0.065
**Place of residence**										
Rural	ref		ref		ref		ref		ref	
Urban	1.57 (1.32, 1.88)	<0.001	0.62 (0.50, 0.77)	<0.001	−6.01 (0.657)	<0.001	−1.30 (0.613)	0.034	0.88 (0.70, 1.11)	0.278

aOR – adjusted odds ratio, β – regression coefficient, CI – confidence interval, ref – reference, SE – standard error

*Logistic regression or linear regressions analysis.

#### Frailty

Frailty was most prevalent among individuals aged ≥70 years (32.3%); women (17.7%); single, divorced, separated, or widowed individuals (20.0%); those with no education (20.6%); and those in the poorest wealth quintile (22.8%). Urban residents (12.2%) had a significantly lower prevalence of frailty compared to rural dwellers (24.2%) (Table S3 in the **Online Supplementary Document**). Adjusted analysis showed that the odds of frailty increased with age and were four times higher among those aged ≥70 years (aOR = 3.98; 95% CI = 3.06, 5.19). Males had lower odds of frailty (aOR = 0.47, 95% CI: 0.37, 0.58) than females. Higher wealth groups had reduced odds of frailty compared to the poorest group. Urban residents had significantly lower odds of frailty than rural residents (aOR = 0.62; 95% CI = 0.50, 0.77). Education level was not significantly associated with frailty (**Table 3**).

#### Disability

Disability worsened with age, with significant increases in disability scores observed in individuals aged ≥70 years compared to those aged 40–49 years. It was also significantly higher in females than in males (Table S3 in the **Online Supplementary Document**). Multivariable results indicated that being aged ≥70 years increased disability by approximately 27 percentage points compared to being aged 40–49 years. Those who were single, divorced, separated, or widowed had disability scores one point higher than those in a partnered relationship. Having a tertiary education, being in higher wealth quintiles, and residing in urban areas were associated with reduced disability compared to individuals with no formal education, those in lower wealth quintiles, and those living in rural areas (**Table 3**).

### Impairments in activities of daily living

Bivariable analysis showed that impairment in activities of daily living varied significantly by age, sex, marital status, education level, and wealth. It was notably higher among individuals aged ≥70 years (48.9%); women (17.3%); single, divorced, separated, or widowed individuals (23.1%); those with no education (22.6%); individuals in the poorest wealth quintile (23.9%); and rural residents (Table S3 in the **Online Supplementary Document**). The multivariable results showed that the odds of ADL impairment increased significantly with age, or more precisely, that they were 20 times higher among individuals aged ≥70 years compared to those aged 40–49 years (aOR = 19.57; 95% CI = 14.42, 26.55). Males had lower odds of ADL impairment than females (aOR = 0.75; 95% CI = 0.60, 0.93). Unexpectedly, having a secondary education was associated with higher odds of ADL impairment (aOR = 1.56; 95% CI = 1.12, 2.17). Individuals from higher wealth groups had lower odds of ADL impairment, while place of residence was not significantly associated with ADL impairment (**Table 3**).

### Quality of life

QoL declined with increasing age and was lower among females and those who reported being single, divorced, separated, or widowed, compared to males and those in partnership relationships (Table S3 in the **Online Supplementary Document**). The multivariable analysis revealed that QoL declined by nearly six percentage points in individuals aged ≥70 years compared to those aged 40–49 years. Being single, divorced, separated, or widowed was associated with a one point decline in QoL. Tertiary education and higher wealth were linked to an increase in QoL, while urban residence was significantly associated with lower QoL (**Table 3**).

## DISCUSSION

We estimated a high prevalence of multimorbidity affecting over half of the study population, exceeding the global estimate of 37.0% and rates reported in other LMICs [52]. Variations in multimorbidity rates across studies may stem from differences in study populations and conditions assessed, with multimorbidity generally being higher in older adults (>60 years) and in studies including more baseline conditions [7]. In Rwanda, the high multimorbidity burden among older adults likely reflects both an ageing population and an epidemiological shift toward noncommunicable diseases. Additionally, the lasting impact of post-genocide trauma, characterised by a high prevalence of posttraumatic stress disorder and major depressive episodes, may have contributed to the increased burden of multimorbidity [31]. These factors collectively pose significant challenges for the existing healthcare system. Increased healthcare utilisation and the need for comprehensive care strategies become critical as the demand for services rises, necessitating a shift towards integrated care models that address the complexities of managing multiple chronic conditions among older Rwandan adults [53].

We found a substantial burden of frailty in this study, with 15.0% of participants classified as frail, lower than some LMIC estimates, such as 72.0% in older adults [54], but higher than the 7.0% prevalence observed in our previous study in rural Burkina Faso [16]. Variations in frailty prevalence across studies may be attributable to differences in populations, frailty assessment tools, and geographical regions [54]. For example, as found in this study and in our previous research of older adults [16,19], frailty increases with age; thus, if older adults are selected, there is a higher likelihood of a higher prevalence of frailty. Nonetheless, evidence suggests that frailty in African settings is modifiable [34]. Interventions like increased physical exercise, health education, and counselling have been shown to delay or reverse frailty in high-income settings [55,56]. Developing and implementing such programmes within Rwanda's primary care system could help slow the progression of frailty in older adults. In contrast to the high burden of multimorbidity and frailty, functional limitation was relatively low, with about 16% of our population reporting functional impairments in basic self-care tasks essential for independent living. This finding may reflect the ability of older Rwandans to maintain physical function, potentially aided by social and community-based support structures. However, moderate quality of life scores in our study may reflect an unmet need or a general impairment in life satisfaction or well-being and thus suggest room for improvement in overall well-being and life satisfaction among older Rwandans.

As expected, our study confirmed age as a critical determinant of health outcomes, with those aged ≥70 years showing significantly higher odds of multimorbidity, frailty, ADL impairments, increased disability, and lower quality of life, aligning with findings from other low-resource settings like Burkina Faso [33]. This is consistent with the biological processes of ageing and the accumulation of lifetime health risks [57]. A notable and concerning finding was the pronounced gender disparity, with men faring better across multiple health outcomes than women. This gender gap corroborates evidence from both LMIC settings [5,6] and high-income countries [58]. Potential explanations for this disparity include biological factors, but also differences in health-seeking behaviour and self-reporting, with women generally having more frequent healthcare consultations and better awareness of their health status than men [59,60]. However, these gender differences may also reflect broader social, economic, and cultural inequities that disadvantage older women in Rwanda. Despite their higher health awareness and more frequent healthcare visits, women often face significant barriers to timely and adequate care, including financial constraints, limited decision-making autonomy, and caregiving responsibilities, all of which exacerbate their vulnerability to poor health outcomes [61–63]. Developing gender-sensitive interventions that address the unique challenges faced by older women, such as policies that improve healthcare accessibility for older women, enhance economic empowerment, and provide targeted mental health and chronic disease management programmes, will be crucial for promoting healthy ageing and achieving more equitable health outcomes. Contrary to our findings, some studies have found no association between multimorbidity and age or gender and frailty, likely due to differing epidemiological profiles [51,64].

Consistent with earlier research [33], marital status also emerged as an important factor, with individuals without a partner demonstrating a higher likelihood of multimorbidity and faring worse in terms of disability and quality of life compared to those with partners. A previous study conducted in the current study setting lends supports to our findings, as it found that being without a partner was significantly associated with the mental and physical dimensions of quality of life [65]. The findings highlight the protective effects of social support and companionship in mitigating health declines. Our finding on the lack of association between marital status and frailty, meanwhile, is supported by a study conducted in Tanzania [66].

Socioeconomic status played a pivotal role in shaping health outcomes, with higher wealth and educational attainment conferring protection against multimorbidity, frailty, disability, and poorer quality of life. These findings reinforce the well-established link between socioeconomic advantages and better health in older age [67–69]. Potential pathways linking higher wealth status to better health outcomes include better access to nutritious food, healthcare, and better living conditions. The educational gradient observed in disability and quality of life scores suggests that individuals with higher education may possess better self-management abilities in older age, possibly due to better health literacy and access to resources [70,71]. However, this trend is not consistent across settings and health outcomes. While this Rwandan population showed better health outcomes among those with higher socioeconomic status, studies in other LMICs have found the opposite pattern, with wealthier individuals showing higher rates of multimorbidity [6,33]. Adding to this complexity, other scholars have found no association between wealth and multimorbidity at all [64]. The higher multimorbidity rates in well-off individuals in some LMIC contexts may be related to less physical activity and consumption of more unhealthy foods, like fats, salt and processed foods [72].

This study also revealed an urban-rural divide, with urban residents experiencing higher levels of multimorbidity and worse quality of life yet reporting less disability (WHODAS) and lower frailty compared to rural residents. Similar patterns have been observed in other low-resource settings, where urban areas tend to carry a higher burden of multimorbidity [6]. Contextually, we believe this urban-rural divide may reflect lifestyle differences, such as diet and prevalence of cardiometabolic conditions, as residing in urban areas is often associated with unhealthier diets [73,74]. In East Africa, including Rwanda, obesity – driven by sedentary lifestyles and unhealthy diets – has become predominantly concentrated in urban settings [75]. While urban residents generally have better access to healthcare services and diagnostic tools, which leads to higher detection rates of chronic conditions, rural residents may underreport their health status due to limited access [76]. Despite higher multimorbidity, urban residents in this Rwandan study demonstrated better disability outcomes and lower frailty prevalence compared to rural residents. This may be due to improved healthcare, assistive technologies, and programmes like car-free days that support physical function and independence in urban areas [77]. However, the worse quality of life reported by urban residents may still reflect social isolation, economic pressures, and stress associated with urban living [78].

This cross-sectional study provides crucial insights into the prevalence and distribution of multimorbidity, frailty, disability, quality of life, and impairment in ADLs among older adults in Rwanda using population-based data from rural and urban areas. By analysing these health outcomes in relation to demographic and socioeconomic factors, we provide the first comprehensive assessment of health and functional ability among Rwanda's ageing population. The findings underscore the substantial health burden facing the ageing population in this East African country, which mirrors trends observed in other LMICs in the region. Importantly, these quantitative findings align closely with older adults’ own expressed priorities, as documented in recent qualitative research in the setting, which found that older people's perception of their needs centred around health and well-being, with a strong emphasis on maintaining physical and mental health [79]. Our quantitative findings complement these expressed needs by providing population-level evidence of the health challenges that older adults face. This integration of quantitative health outcomes with older adults' lived experiences and priorities creates a more comprehensive framework for intervention. Healthcare providers and policymakers can use this combined understanding to develop targeted programmes that not only address clinical needs but also respond to older adults' desires for a supportive environment that enables healthy and active ageing. This evidence-based, person-centred approach is particularly crucial as Rwanda continues to strengthen its healthcare system and adapt to the growing needs of its ageing population.

### Strengths and limitations

A key strength of our study is the large sample of over 4300 older adults, which enhances statistical power, generalisability, and allows for precise estimates, meaningful subgroup analyses, and robust multivariable modelling. However, several limitations should also be considered when interpreting these findings. First, the cross-sectional design limits our ability to infer causality between the exposures and the outcome. However, the findings provide a foundation for future longitudinal studies that could explore the temporal dynamics of multimorbidity and frailty in older Rwandans. Second, the study’s one-year residency requirement may exclude certain vulnerable populations, such as seasonal workers and internally displaced individuals, who could be at higher risk for multimorbidity, frailty, and disability. While this criterion ensures that participants have adequate exposure to local healthcare systems and their village environment, it also limits the generalisability of our findings. Third, some conditions were diagnosed using biological criteria, whereas others were based on self-reported symptoms and thus may result in either an underestimation or overestimation of the population prevalence. However, while self-reported data may introduce some recall bias, the use of validated tools and objective measurements for assessing health outcomes strengthens the credibility of some of our measures, including frailty, disability, and quality of life. For example, we assessed quality of life using the eight-item EUROHIS-WHOQoL scale. Although this index is unidimensional and may not capture the full range of quality of life as comprehensively as the 26-item WHOQoL-Abbreviated Version (WHOQoL-BREF), its psychometric properties are comparable to those of the WHOQoL-BREF, rendering it a valid and reliable tool for assessing quality of life [44]. Additionally, the duration of the disease and treatment could influence the outcomes and impact of comorbidities. Unfortunately, the dataset used in this analysis did not include this information, which may have provided a more comprehensive understanding of the effects of comorbidities. Finally, height measurements were obtained using a tape measure, which may introduce minor errors due to posture variations and inter-observer differences. Although this method is commonly used in field settings, more precise alternatives, such as stadiometers, could improve measurement accuracy.

## CONCLUSIONS

The high prevalence of multimorbidity, frailty, and disability among older adults in Rwanda calls for urgent healthcare system reforms. Integrated care models that address both the physical and social determinants of health, with a focus on reducing gender, socioeconomic, and geographical disparities, are needed to improve the well-being of older populations. The higher burden of poor health outcomes among women further indicates the need for gender-sensitive health interventions to address the unique health challenges faced by older women in Rwanda. Implementing these strategies within Rwanda's primary care system and social support programmes could significantly enhance healthy ageing and universal health coverage in the country. Our findings can inform the development of national policies and targeted interventions to promote active and successful ageing in Rwanda.

## Additional material


Online Supplementary Document
