# Supplementary material for: Health and well-being of older adults in rural and urban Rwanda: epidemiological findings from a population based cross-sectional study
Source: J Glob Health. 2025 May 5;15:04108. doi: 10.7189/jogh.15.04108 (PMC12050114; doi:10.7189/jogh.15.04108)
Supplement: Online Supplementary Document [file jogh-15-04108-s001.pdf]

Supplement to: Boah M, Cyuzuzo C, Uwinkindi F, Kalinda C, Yohannes T, Isano S, Greig C, Davies J, Hirschorn LR, Amberbir A. Health and wellbeing of older adults in rural and urban Rwanda: epidemiological findings from a population based cross-sectional study. J Glob Health. 2025;15:04108.

Table S1: Gender differences in chronic conditions and health outcomes, including multimorbidity, frailty, quality of life, and disability among adults (n = 4369)

**Outcome**	**Categories**	**Total (n = 4369) n (%)**	**Female (n = 2757) n (%)**	**Male (n =1612) n (%)**	***p-value***
**Noncommunicable conditions**	Hypertension	1751 (40.1)	1144 (41.5)	607 (37.7)	0.012
	High cholesterol	43 (1.0)	26 (1.0)	17 (1.1)	0.710
	Raised blood glucose	365 (8.4)	238 (8.6)	127 (7.9)	0.380
	Heart disease	84 (1.9)	55 (2.0)	29 (1.8)	0.650
	Stroke	657 (15.0)	427 (15.5)	230 (14.3)	0.280
	Chronic respiratory disease	243 (5.6)	161 (5.9)	82 (5.1)	0.290
	Cancer	69 (1.6)	47 (1.7)	22 (1.4)	0.390
	Musculoskeletal conditions (e.g., bone, joint)	1277 (29.2)	913 (33.1)	364 (22.6)	0.000
	Symptoms of anxiety on testing	1395 (31.9)	968 (35.1)	427 (26.5)	0.000
	Depressive symptoms (≥ 10 points)	1018 (23.3)	702 (25.5)	316 (19.6)	0.000
	No depressive symptoms (0-4)	1907 (43.6)	1082 (39.2)	825 (51.2)	0.001
	Mild depressive symptoms (5-9)	1444 (33.1)	973 (35.3)	471 (29.2)	
	Moderate depressive symptoms (10-14)	652 (14.9)	453 (16.4)	199 (12.3)	
	Moderately severe depressive symptoms (15-19)	252 (5.8)	173 (6.3)	79 (4.9)	
	Severe depressive symptoms (20-27)	114 (2.6)	76 (2.8)	38 (2.4)	
	Dementia absent	3869 (88.6)	2471 (89.6)	1398 (86.7)	0.015
	Probable dementia	124 (2.8)	71 (2.6)	53 (3.3)	
	Possible dementia	376 (8.6)	215 (7.8)	161 (10.0)	
	Symptoms of cognitive impairment (probable or possible dementia)	500 (11.4)	286 (10.4)	214 (13.3)	0.004
	Chronic eye conditions (e.g. cataract, glaucoma)	757 (17.3)	526 (19.1)	231 (14.3)	0.000
	Chronic kidney disease	67 (1.5)	39 (1.4)	28 (1.7)	0.400
	Chronic liver diseases	28 (0.6)	18 (0.7)	10 (0.6)	0.900
**Chronic communicable diseases**	HIV infection	308 (9.3)	212 (10.3)	96 (7.7)	0.011
**Multimorbidity (any 2 or more chronic conditions)**	No	1959 (44.8)	1128 (40.9)	831 (51.6)	0.000
	Yes	2410 (55.2)	1629 (59.1)	781 (48.4)	
**Nature of multimorbidity**	Concordant multimorbidity	1192 (27.3)	794 (28.8)	398 (24.7)	0.003
	Discordant multimorbidity	1218 (27.9)	835 (30.3)	383 (23.8)	0.000

Outcome	Categories	Total<br>(n = 4369)<br>n (%)	Female<br>(n = 2757)<br>n (%)	Male (n =1612)<br>n (%)	*p-value*
**Domains of multimorbidity**	Cardiometabolic conditions	553 (12.7)	366 (13.3)	187 (11.6)	0.108
	Mental health conditions	798 (18.3)	544 (19.7)	254 (15.8)	0.001
**Frailty**	Not frail	1340 (30.7)	716 (26.0)	624 (38.7)	0.000
	Pre-frail	2394 (54.8)	1552 (56.3)	842 (52.2)	
	Frail/unable to score	635 (14.5)	489 (17.7)	146 (9.1)	
**Quality of life**	WHOQoL score (0-100): mean (SD)	48.2 (±15.6)	47.6 (± 15.2)	49.2 (±16.3)	0.000
**Disability**	WHODAS 2.0 score (0-100): median (IQR)	10.4 (2.1-25.0)	12.5 (4.2-29.2)	6.2 (0.0-18.7)	0.000
**Impairment in activities of daily living (ADL)**	None	3671 (84.0)	2279 (82.7)	1392 (86.4)	0.001
	Some impairment (≥1 ADL)	698 (16.0)	478 (17.3)	220 (13.6)	
**Impairment severity (among individuals reporting impairment in ADL; n= 698)**	Mild	376 (53.9)	260 (54.4)	116 (52.7)	0.486
	Moderate to severe	265 (38.0)	183 (38.3)	82 (37.3)	
	Extreme	57 (8.2)	35 (7.3)	22 (10.0)	

p – values are obtained from chi-square tests, t-tests, ANOVA, Mann-Whitney U test, or Kruskal-Wallis H test, as applicable

Table S2: Rural urban differentials in the prevalence of chronic conditions and health outcomes, including multimorbidity, frailty, quality of life, and disability among adults (n = 4369)

Outcome	Categories	Total (n = 4369) n (%)	Rural (n = 833) n (%)	Urban (n =3536) n (%)	*p-value*
**Noncommunicable conditions**	Hypertension	1751 (40.1)	295 (35.4)	1456 (41.2)	0.002
	High cholesterol	43 (1.0)	1 (0.1)	42 (1.2)	0.006
	Raised blood glucose	365 (8.4)	41 (4.9)	324 (9.2)	0.000
	Heart disease	84 (1.9)	35 (4.3)	49 (1.4)	0.000
	Stroke	657 (15.0)	174 (20.9)	483 (13.7)	0.000
	Chronic respiratory disease	243 (5.6)	20 (2.4)	223 (6.3)	0.000
	Cancer	69 (1.6)	9 (1.1)	60 (1.7)	0.200
	Musculoskeletal conditions	1277 (29.2)	199 (23.9)	1078 (30.5)	0.000
	Symptoms of anxiety on testing	1395 (31.9)	295 (35.4)	1100 (31.1)	0.016
	Depressive symptoms (≥ 10 points)	1018 (23.3)	325 (39.0)	693 (19.6)	0.000
	No depressive symptoms (0-4)	1907 (43.6)	210 (25.2)	1697 (48.0)	0.000
	Mild depressive symptoms (5-9)	1444 (33.1)	298 (35.8)	1146 (32.4)	
	Moderate depressive symptoms (10-14)	652 (14.9)	171 (20.5)	481 (13.6)	
	Moderately severe depressive symptoms (15-19)	252 (5.8)	107 (12.8)	145 (4.1)	
	Severe depressive symptoms (20-27)	114 (2.6)	47 (5.6)	67 (1.9)	
	Dementia absent	3869 (88.6)	707 (84.9)	3162 (89.4)	0.001
	Probable dementia	124 (2.8)	32 (3.8)	92 (2.6)	
	Possible dementia	376 (8.6)	94 (11.3)	282 (8.0)	
	Symptoms of cognitive impairment (probable or possible dementia)	500 (11.4)	126 (15.1)	374 (10.6)	0.000
	Chronic eye conditions (e.g. cataract, glaucoma)	757 (17.3)	92 (11.0)	665 (18.8)	0.000
	Chronic kidney disease	67 (1.5)	14 (1.7)	53 (1.5)	0.700
	Chronic liver diseases	28 (0.6)	5 (0.6)	23 (0.7)	0.870
**Chronic communicable diseases**	HIV infection	308 (9.3)	17 (3.9)	291 (10.2)	0.000
**Multimorbidity (any 2 or more chronic conditions)**	No	1959 (44.8)	390 (46.8)	1569 (44.4)	0.200
	Yes	2410 (55.2)	443 (53.2)	1967 (55.6)	
	Concordant multimorbidity	1192 (27.3)	285 (34.2)	907 (25.7)	0.000

Outcome	Categories	Total (n = 4369) n (%)	Rural (n = 833) n (%)	Urban (n =3536) n (%)	*p-value*
**Nature of multimorbidity**	Discordant multimorbidity	1218 (27.9)	158 (19.0)	1060 (30.0)	0.000
**Domains of multimorbidity**	Cardiometabolic conditions	553 (12.7)	105 (12.6)	448 (12.7)	0.960
	Mental health disorders	798 (18.3)	232 (27.9)	566 (16.0)	0.000
**Frailty**	Not frail	1340 (30.7)	143 (17.2)	1197 (33.9)	0.000
	Pre-frail	2394 (54.8)	488 (58.6)	1906 (53.9)	
	Frail/unable to score	635 (14.5)	202 (24.2)	433 (12.2)	
**Quality of life**	WHOQoL score (0-100): mean (SD)	48.2 (± 15.6)	45.0 (± 14.9)	49.0 (±15.7)	0.000
**Disability**	WHODAS 2.0 score (0-100): median (IQR)	10.4 (2.1-25.0)	20.8 (6.2-41.7)	8.3 (2.1-20.8)	0.000
**Impairment in activities of daily living (ADL)**	None	3671 (84.0)	637 (76.5)	3034 (85.8)	0.000
	Some impairment (≥1 ADL)	698 (16.0)	196 (23.5)	502 (14.2)	
**Impairment severity (among individuals reporting impairment in ADL; n= 698)**	Mild	376 (53.9)	93 (47.4)	283 (56.4)	0.089
	Moderate to severe	265 (38.0)	83 (42.3)	182 (36.3)	
	Extreme	57 (8.2)	20 (10.2)	37 (7.4)	

p-values from chi-square tests, student t-test, ANOVA, Mann-Whitney U test, or Kruskal-Wallis H test, as applicable

Table S3: Distribution of multimorbidity, frailty status, quality of life, disability, and impairment in activities of daily living by respondents' background characteristics

Variable	Category	Multimorbidity % (95% CI)	Frail % (95 % CI)	Disability Median (IQR)	WHOQoL Mean (SD)	Impairment in ADLs % (95% CI)
**Age group (years)**	40-49	41.4 (39.1, 43.8)	9.3 (8.0, 10.7)	4.2 (0.0-12.5)	50.5 (14.5)	4.3 (3.4, 5.4)
	50-59	53.4 (50.5, 56.2)	9.0 (7.5, 10.8)	9.4 (2.1-20.8)	48.9 (15.3)	10.8 (9.2, 12.7)
	60-69	66.6 (63.4, 69.7)	18.6 (16.2, 21.4)	18.7 (4.2-33.3)	46.9 (16.1)	20.4 (17.8, 23.2)
	70+	77.8 (74.5, 80.8)	32.3 (28.8, 35.9)	37.5 (18.7-56.2)	42.9 (16.9)	48.9(45.1, 52.7)
	p-value	*0.000*	*0.000*	*0.000*	*0.000*	*0.000*
**Sex**	Female	59.1 (57.2, 60.9)	17.7 (16.4, 19.2)	12.5 (4.2-29.2)	47.6 (15.2)	17.3 (16.0, 18.8)
	Male	48.4 (46.0, 50.9)	9.1 (7.8. 10.6)	6.2 (0.0-18.7)	49.3 (6.7)	13.6 (12.1, 15.4)
	p-value	0.000	0.000	0.000	0.001	0.001
**Marital status**	Married/cohabiting	48.9 (47.0, 50.7)	11.5 (10.4, 12.7)	6.2 (0-18.7)	49.9 (15.4)	12.0 (10.9, 13.3)
	Single/divorced/ separated/widowed	66.6 (64.3, 69.0)	20.0 (18.1, 22.1)	16.7 (4.2-33.3)	45.3 (15.5)	23.1 (21.11, 25.3)
	p-value	*0.000*	*0.000*	*0.000*	*0.000*	*0.000*
**Educational level**	No education	60.3 (58.0, 62.6)	20.6 (18.8, 22.6)	16.7 (4.2-35.4)	45.0 (15.7)	22.6 (20.8, 24.7)
	Primary	52.0 (49.8, 54.2)	10.3 (9.0, 11.7)	8.3 (0.0-18.7)	49.6 (14.8)	10.6 (9.3, 12.0)
	Secondary	52.7 (48.3, 57.1)	12.0 (9.4, 15.2)	6.3 (0.0-20.8)	51.3 (15.7)	16.3 (13.3, 19.8)
	Tertiary	40.9 (32.1, 50.3)	5.5 (2.5, 11.6)	2.1 (0.0-10.4)	61.5 (13.0)	5.5 (2.5, 11.6)
	p-value	*0.000*	*0.000*	*0.000*	*0.000*	*0.000*
**Wealth quintile**	Poorest	63.4 (60.1, 66.5)	22.8 (20.1, 25.7)	18.7 (4.2-37.5)	39.8 (16.2)	23.9 (21.2, 26.9)
	Poorer	56.4 (53.1, 59.7)	16.3 (14.0, 18.8)	14.6 (4.2-29.2)	46.3 (14.1)	18.0 (15.6, 20.7)
	Middle	52.5 (49.2, 55.8)	12.0 (10.0, 14.3)	10.4 (2.1-22.9)	48.1 (13.7)	12.8 (10.7, 15.1)
	Richer	51.3 (48.0, 54.6)	12.7 (10.6, 15.1)	6.2 (0.0-18.7)	50.9 (14.5)	10.5 (8.6, 12.7)
	Richest	52.2 (48.9, 55.5)	9.0 (7.3, 11.1)	6.2 (0.0-18.7)	56.0 (14.8)	14.8 (12.5, 17.3)
	p-value	*0.000*	*0.000*	*0.000*	*0.000*	*0.000*
**Place of residence**	Rural	53.2 (49.8, 56.5)	24.2 (21.5, 27.3)	20.8 (6.2-41.7)	45.0 (14.9)	23.5 (20.8, 26.5)
	Urban	55.6 (54.0, 57.3)	12.2 (11.2, 13.4)	8.3 (2.1-20.8)	49.0 (15.9)	14.2 (13.1, 15.4)
	p-value	*0.201*	*0.000*	*0.000*	*0.000*	*0.000*

ADLs: activities of daily living; CI: confidence interval; IQR: interquartile range; SD: standard deviation; WHOQoL: World Health Organization Quality of Life. *p-values from chi-square tests, student t-test, ANOVA, Mann-Whitney U test or Kruskal-Wallis H test, as applicable*

Table S4: Prevalence rates of multimorbidity of cardiometabolic conditions, mental health multimorbidity, and concordant and discordant multimorbidity across respondents' background characteristics

Variable	Multimorbidity of cardio-metabolic conditions % (95% CI)	Multimorbidity of mental health conditions % (95% CI)	Concordant multimorbidity % (95% CI)	Discordant multimorbidity % (95% CI)
**Age groups**				
40-49	6.91(5.78, 8.23)	11.4 (10.0, 13.0)	17.06 (15.33, 18.94)	24.38 (22.38, 26.51)
50-59	11.82 (10.10, 13.79)	13.2 (11.4, 15.2)	22.61 (20.32, 25.10)	30.78 (28.21, 33.48)
60-69	17.92 (15.50, 20.62)	21.7 (19.1, 24.6)	35.14 (32.03, 38.39)	31.45 (28.44, 34.62)
70+	21.72 (18.74, 25.02)	40.0 (36.3, 43.7)	50.98 (47.18, 54.77)	26.85 (23.61, 30.35)
p-value	*0.000*	*0.000*	*0.000*	*0.000*
**Sex**				
Female	13.28 (12.06, 14.59)	19.7 (18.3, 21.3)	28.80 (27.14, 30.52)	30.29 (28.60, 32.03)
Male	11.60 (10.13, 13.26)	15.8 (14.1, 17.6)	24.69 (22.65, 26.86)	23.76 (21.74, 25.90)
p-value	*0.108*	*0.001*	*0.003*	*0.000*
**Marital status**				
Married/cohabiting	11.69 (10.56, 12.93)	14.9 (13.7, 16.3)	23.85 (22.31, 25.46)	25.02 (23.45, 26.65)
Single/divorced/separated/widowed	14.41 (12.75, 16.26)	24.3 (22.2, 26.5)	33.55 (31.24, 35.94)	33.10 (30.79, 35.48)
p-value	*0.010*	*0.000*	*0.000*	*0.000*
**Educational level**				
No education	13.07 (11.58, 14.73)	25.1 (23.2, 27.2)	32.48 (30.34, 34.71)	27.84 (25.80, 29.98)
Primary	11.19 (9.89, 12.65)	13.7 (12.3, 15.3)	23.19 (21.39, 25.09)	28.79 (26.84, 30.81)
Secondary	16.29 (13.28, 19.83)	15.7 (12.7, 19.2)	27.49 (23.72, 31.62)	25.25 (21.61, 29.29)
Tertiary	16.36 (10.56, 24.49)	1.8 (0.5, 7.0)	17.27 (11.30, 25.50)	23.64 (16.62, 32.46)
p-value	*0.010*	*0.000*	*0.000*	*0.323*
**Wealth quintile**				
Poorest	9.27 (7.52, 11.38)	34.2 (31.1, 37.4)	38.22 (35.05, 41.48)	25.17 (22.40, 28.16)
Poorer	12.70 (10.65, 15.08)	20.4 (17.8, 23.2)	28.15 (25.26, 31.22)	28.26 (25.37, 31.34)
Middle	12.53 (10.51, 14.88)	15.7 (13.4, 18.2)	25.06 (22.31, 28.02)	27.43 (24.59, 30.46)
Richer	12.11 (10.10, 14.46)	11.5 (9.6, 13.8)	21.34 (18.74, 24.19)	29.99 (27.03, 33.13)
Richest	16.71 (14.37, 19.34)	9.5 (7.7, 11.6)	23.61 (20.91, 26.56)	28.57 (25.66, 31.67)
p-value	*0.000*	*0.000*	*0.000*	*0.243*
**Place of residence**				
Rural	12.61 (10.52, 15.04)	27.8 (24.9, 31.0)	34.21 (31.07, 37.51)	18.97 (16.45, 21.77)
Urban	12.67 (11.61, 13.81)	16.0 (14.8, 17.2)	25.65 (24.24, 27.12)	29.98 (28.49, 31.51)
p-value	*0.960*	*0.000*	*0.000*	*0.000*

p-values from chi-square tests, student t-test, ANOVA, Mann Whitney test, or Kruskal-Wallis H test, as applicable

Table S5: Results of Tukey's Honest Significant Difference (HSD) post-hoc test

Variable	Comparison groups	Contrast (Mean difference)	95% CI		*p*-value
**Age groups, years**	50-59 vs 40-49	-1.57	-3.08	-0.07	0.036
	60-69 vs 40-49	-3.61	-5.27	-1.95	0.000
	70-79 vs 40-49	-7.59	-9.41	-5.77	0.000
	60-69 vs 50-59	-2.03	-3.80	-0.26	0.017
	70-79 vs 50-59	-6.01	-7.94	-4.09	0.000
	70-79 vs 60-69	-3.98	-6.02	-1.94	0.000
**Educational level**	Primary vs No education	4.69	3.4	6.0	0.000
	Secondary vs No education	6.35	4.3	8.4	0.000
	Tertiary vs No education	16.50	12.6	20.4	0.000
	Secondary vs Primary	1.66	-0.3	3.6	0.135
	Tertiary vs Primary	11.81	8.0	15.7	0.000
	Tertiary vs Secondary	10.15	6.0	14.3	0.000
**Wealth quintile**	Poorer vs Poorest	6.50	4.6	8.4	0.000
	Middle vs Poorest	8.32	6.4	10.2	0.000
	Richer vs Poorest	11.08	9.2	13.0	0.000
	Richest vs Poorest	16.17	14.3	18.1	0.000
	Middle vs Poorer	1.81	-0.1	3.7	0.072
	Richer vs Poorer	4.57	2.7	6.5	0.000
	Richest vs Poorer	9.67	7.8	11.6	0.000
	Richer vs Middle	2.76	0.8	4.7	0.001
	Richest vs Middle	7.86	5.9	9.8	0.000
	Richest vs Richer	5.10	3.2	7.0	0.000
